# Reliability of pressure-volume loop parameters derived from transthoracic echocardiography in patients undergoing hemodialysis

**DOI:** 10.1371/journal.pone.0340206

**Published:** 2026-01-05

**Authors:** Dinara Galiyeva, Zaukiya Khamitova, Aizhan Zhankorazova, Ayan Nurkesh, Dinara Jumadilova, Bauyrzhan Toktarbay, Murat Mukarov, Alexey Kokoskho, Abduzhappar Gaipov, Alessandro Salustri

**Affiliations:** 1 Department of Medicine, Nazarbayev University School of Medicine, Astana, Kazakhstan; 2 Heart Center University Medical Center (UMC), Astana, Kazakhstan; 3 Astana Medical University, Astana, Kazakhstan; 4 Clinical Academic Department of Internal Medicine, University Medical Center (UMC), Astana, Kazakhstan; Scuola Superiore Sant'Anna, ITALY

## Abstract

**Background:**

The pressure-volume (PV) relationship remains a key tool for assessing cardiac function. PV loop derived parameters, including end-systolic elastance (Ees), effective arterial elastance (Ea), ventriculo-arterial coupling (VAC), stroke work (SW), pressure-volume area (PVA), and work efficiency (WE), are valuable tools, but reproducibility data are scarce.

**Objective:**

To evaluate the inter- and intra-observer variability of PV loop parameters derived from transthoracic echocardiography images in patients undergoing hemodialysis.

**Methods:**

Twenty-five adult patients with end-stage renal disease undergoing maintenance hemodialysis were randomly selected. PV loops were reconstructed using Q-Strain software (version 1.3.0.79, Medis, Leiden, the Netherlands), and parameters including Ees, Ea, VAC, SW, PVA, and WE were calculated. Two experienced readers performed measurements for inter-observer analysis; one reader repeated measurements one week apart for intra-observer analysis. Reliability was assessed using intraclass correlation coefficients (ICC) and Bland-Altman plots.

**Results:**

Inter-observer agreement was excellent for SW 0.96 (0.91–0.98) and PVA 0.95 (0.87–0.98), good for Ees 0.90 (0.77–0.96) and Ea 0.79 (0.49–0.91), and moderate for VAC 0.68 (0.23–0.85) and WE 0.72 (0.28–0.79). Intra-observer agreement was excellent for Ees 0.96 (0.90–0.98), Ea 0.98 (0.94–0.99), SW 0.98 (0.96–0.99) and PVA 0.98 (0.96–0.99), and good for VAC 0.78 (0.51–0.91) and WE 0.85 (0.65–0.94). Bland-Altman analysis showed minimal bias for Ees, SW and PVA, whereas Ea, VAC and WE exhibited proportional bias.

**Conclusions:**

PV loop-derived parameters obtained via transthoracic echocardiography demonstrate good-to-excellent reproducibility, particularly for Ees, Ea, SW and PVA. VAC and WE show moderate variability, suggesting careful interpretation of these parameters in clinical and research settings.

## Introduction

The pressure-volume (PV) relationship, introduced by Otto Frank in 1895, continues to play an essential role in cardiovascular medicine as a powerful tool for analysing cardiac function [1]. Several parameters derived from the PV loop – such as end-systolic elastance (Ees), effective arterial elastance (Ea), ventriculo-arterial coupling (VAC), stroke work (SW), pressure-volume area (PVA), and work efficiency (WE) – provide detailed insights into myocardial contractility, arterial load, and overall cardiovascular performance [1–3]. In particular, VAC has been recognized as one of the key parameters to accurately evaluate the hemodynamic relationship between ventricular contractility and the arterial system, and it has been extensively investigated in patients with heart failure (HF), where it reflects the interaction between myocardial contractility and arterial load. Altered VAC is associated with impaired cardiac efficiency, adverse outcomes, and may guide therapeutic decisions [4,5].

Patients undergoing maintenance hemodialysis (HD) often exhibit marked cardiovascular alterations, including pressure and volume shifts, myocardial remodeling, and increased arterial stiffness [6,7]. In such patients, conventional parameters of left ventricular function often lack sensitivity and specificity. Therefore, reliable non-invasive measurement of PV loop parameters could offer a superior approach to understanding cardiac function in this very high-risk population [8,9]. Prior studies have examined reproducibility of PV loop parameters derived from cardiac magnetic resonance imaging [10,11], however the reproducibility and reliability in patients on HD have not been evaluated yet. Therefore, it remains unclear whether PV loop measurements derived from transthoracic echocardiography (TTE) are consistent and clinically applicable in HD patients.

To address this gap, the present study aimed to evaluate the inter- and intra-observer variability of TTE-derived PV loop parameters in patients undergoing HD. By clarifying the reproducibility of these indices, we sought to support their potential use in both clinical practice and research focused on cardiovascular assessment in HD patients.

## Materials and methods

### Study population

This study was conducted as part of a larger project investigating the impact of hemodialysis-induced volume changes on PV loop, derived from speckle-tracking echocardiography in patients with end-stage renal disease [9]. For the purpose of this study, 25 adult patients were randomly selected. The study design and procedures followed the protocol previously published [9]. In brief, we included adult patients (≥18 years) undergoing maintenance HD on a standard thrice-weekly schedule for at least 3 months, with dialysis adequacy defined as single-pool Kt/V > 1.2 according to international guidelines. Exclusion criteria were: prior kidney transplantation; myocardial infarction, unstable angina, or stroke within the preceding 6 months; severe (stage III–IV) HF according to the New York Heart Association classification; permanent atrial fibrillation; body mass index >40 kg/m²; malignancy or other conditions associated with poor prognosis; and nonadherence to the prescribed dialysis schedule during the previous month. The main reasons for applying these criteria were to avoid confounding factors that may affect the hemodynamic parameters and to exclude patients with unstable conditions.

For each participant, we recorded demographic and clinical variables including age, sex, dialysis vintage, body mass index, blood pressure, heart rate, comorbidities and medications. The study was approved by the Institutional Research Ethics Committee of Nazarbayev University (NU-IREC #550/20042022) and all participants provided written informed consent.

### Data collection

As described in the published study protocol [9], TTE was performed before the dialysis session using standardized acquisition settings. All images were digitally stored and analyzed offline by two independent observers blinded to clinical data and to each other’s measurements. For intra-observer analysis, repeated measurements were performed by the same observer after a one-week interval, while inter-observer variability was assessed by comparing measurements obtained independently by two readers.

### Echocardiography acquisition

TTE images were acquired by an experienced sonographer using a commercially available ultrasound system (Philips CX50 equipped with a transducer S5-1, Philips, The Netherlands). Apical 4-chamber, 2-chamber, and long-axis views were obtained for the visualization of the left ventricular (LV) endocardial border. The images were digitally stored and transferred on an external driver for further analysis.

### Echocardiography analysis

Offline analysis was performed using Q-Strain software (version 1.3.0.79, Medis, Leiden, the Netherlands) as described previously [12]. Two independent readers (DJ and AZ) with significant experience in cardiac imaging using this software tool performed the measurements. After selecting end-diastolic and end-systolic frames in the 2-, 3-, and 4-chamber views, observers traced the LV endocardial borders using a semi-automated tool with manual adjustments as needed. The LV outflow tract, papillary muscles, and trabeculae were included in the cavity. Modified biplane Simpson’s method was used to evaluate the end-diastolic and end-systolic volumes, and the derived LV ejection fraction. Patient-specific brachial systolic pressure and time–volume data were then integrated by the software to reconstruct the PV loop based on a previously described model [3].

### PV loop reconstruction

**Fig 1:**
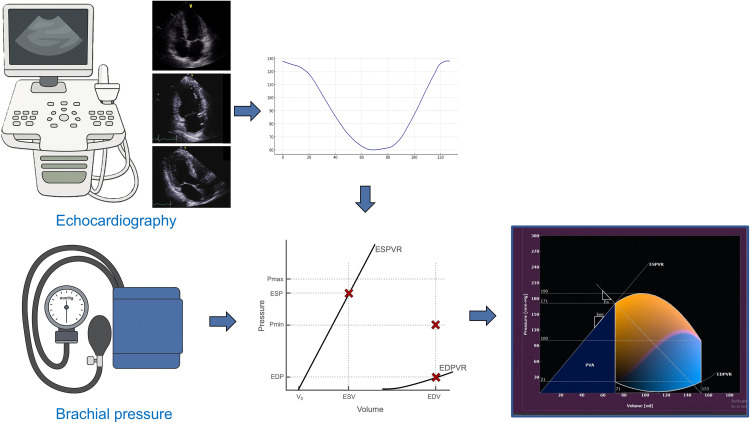
Workflow for pressure–volume (PV) loop reconstruction using transthoracic echocardiography. Left ventricular endocardial contours are traced in the 2-, 3-, and 4-chamber views. From these data, volume–time curves are derived and combined with patient-specific brachial systolic pressure. Dedicated software applies a physics-based model to integrate imaging-derived volumes with cuff-measured pressure and reconstruct the end-systolic and end-diastolic PV relations.

The PV loop was reconstructed according to the approach of Pedrizzetti et al. [3]. In brief, the algorithm defined the end-systolic and end-diastolic PV relationships (ESPVR and EDPVR). The ESPVR was expressed as P = Ees (V − V_0_), where ventricular elastance (Ees) was calculated from the LV end-systolic volume (VES) and the estimated end-systolic pressure (PES, assumed to be 90% of brachial systolic pressure) [13]. The intercept volume V_0_ was obtained as V_0_ = VES – (PES/Ees). The EDPVR was modeled as P = αVβ, with the parameters α and β derived from end-diastolic volume (EDV) and end-diastolic pressure (EDP). Patient-specific inputs (LV volume–time data and brachial systolic pressure) were then combined within the algorithm, and the resulting PV loop represented one complete cardiac cycle with its characteristic phases: isovolumic contraction, ejection, isovolumic relaxation, and diastolic filling. The flowchart of the PV loop reconstruction is represented in Fig 1.

### PV loop analysis

**Fig 2:**
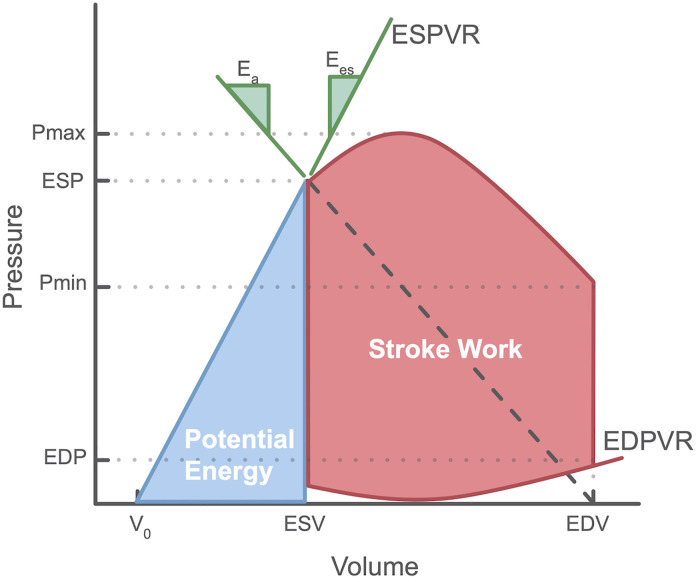
Pressure-Volume (PV) loop of the left ventricle: the graphical representation of the pressure and volume relationship in the left ventricle during cardiac cycle with the derived parameters: V_0_, theoretical volume at zero pressure; ESV/EDV, end-systolic and end-diastolic volume; ESP/EDP, end-systolic and end-diastolic pressure; ESPVR/EDPVR, end-systolic and end-diastolic pressure-volume relationships; Pmin/Pmax, minimum and maximum brachial pressure; Ea, effective arterial elastance; Ees, end-systolic elastance; PE, potential energy; PVA, pressure volume area; SW, stroke work; WE, work efficiency.

The reconstructed PV loop shown in Fig 2 was analyzed to derive the following hemodynamic parameters:

- End-systolic elastance (Ees): It expresses the left ventricular contractility as the ESPVR slope
- Effective arterial elastance (Ea): The EDPVR slope provides the value for the effective arterial afterload
- Ventriculo-arterial coupling (VAC): calculated by the Ea/Ees ratio
- Stroke work (SW): the area enclosed by the PV loop
- Pressure volume area (PVA): the sum of the potential energy and stroke work
- Work efficiency (WE): the ratio of SW over PVA

A detailed description of these parameters and their derivation has been reported previously [3,9,14].

### Statistical methods

The differences between repeated measurements by one rater (intra-observer) and between raters (inter-observer) were assumed to be normally distributed. The bias, agreement, and the association between groups were analyzed following established protocols [15]. Stata 18.0 (StataCorp, College Station, TX) software was used for the statistical analysis.

#### Association.

*Intraclass correlation coefficient*. The intraclass correlation coefficient (ICC) and its 95% confidence interval (CI) were used to evaluate the reliability of consistency of measurements made on the same 25 patients. A two-way mixed effect model was applied. For intra-observer reliability (repeated measurements by the same operator over 1 week), a single-rating, absolute agreement ICC was used. For inter-observer reliability (measurement from two different operators), the mean-rating (k = 2), absolute agreement ICC was applied [16]. The ICC value ranges from 0 to 1, where values below 0.50 indicate poor reliability, between 0.50 and 0.75 moderate reliability, between 0.75 and 0.90 good reliability, and above 0.90 excellent reliability. A p-value <0.05 was considered statistically significant.

#### Bias and agreement.

*Bland-Altman plot*. The degree of agreement between different measurements was assessed by constructing the Bland-Altman plots. At this aim, 1) the difference in the pair of measurements were plotted against their averages, 2) the bias (the mean of the differences) were calculated and plotted, and 3) the upper and lower limits of agreement (LoA) (bias + /- 1.96 x SD of difference) were calculated and plotted. The LoA define the range within which the majority of measurement differences fall. The interpretation of the Bland-Altman is based on the clinical relevance of both the bias and the width of LoA. A higher level of agreement is indicated when the differences are clustered around zero. If the differences consistently deviate from zero, this reflects a systematic bias, showing a continuous difference in measurements between the observers. In addition, a linear regression line was fitted to the differences versus the averages to assess the proportional bias, with a statistically significant slope (for p-value<0.05) indicating the magnitude of disagreement changes depending on the size of the measured value.

#### Sample size calculation.

In order to find the statistically significant sample size needed to recognize relative changes in PV loop parameters (90% power and 5% significance threshold), the following formula was used [17–19]:


$$n\;=\;f(\alpha ,P)\times \sigma^{2}\times \frac{2}{\delta^{2}}$$

where *n* stands for sample size, α for the significance level, *P* for the study power required, *f* the value of the factor for different values of α and *P* (*f* = 10.5 for α = 0.05 and p = 0.090), and δ the desired difference to be detected. Standard deviation for the calculations between separate two studies is abbreviated as σ. The above calculations were performed for the inter-observer variability numbers.

## Results

Data underlying the findings described in this article are reported in the S1 File. Demographic and clinical characteristics of the patients are summarized in Table 1. The study included 25 HD patients with a mean age of 52.6 (SD 17.9) years, of whom 76% were male. The average body mass index was 23.4 (SD 3.8) kg/m², and the median dialysis vintage was 21 months (IQR 13.0–68.0). Hypertension was the most common comorbidity (88%), followed by diabetes (24%), HF (16%) and history of coronary artery disease (4%) or revascularization (4%). None of the patients presented with left bundle branch block, cardiac resynchronization therapy, severe chronic obstructive pulmonary disease, or hemodynamically significant valvular heart disease. Regarding medications, 20% of the patients were on ACE inhibitors or ARBs, 16% on beta-blockers, 28% on calcium channel blockers, and 4% on phosphate binders.

**Table 1:** Demographic and clinical characteristics of the study group (n = 25).

Parameter	
Age, years (SD)	52.6 (17.9)
Male, n (%)	19 (76.0)
Height, cm (SD)	170.4 (6.5)
BMI, kg/m^2^ (SD)	23.4 (3.8)
BSA, m^2^ (SD).	1.78 (0.18)
HR, bpm (SD)	71.4 (1.9)
SBP, mmHg (SD)	153.2 (23.4)
DBP, mmHg (SD)	93.6 (16.3)
MAP, mmHg (SD)	113.5 (16.7)
**Comorbidity**	
Hypertension, n (%)	22 (88.0)
Diabetes, n (%)	6 (24.0)
Smoking history, n (%)	2 (8.0)
Coronary artery disease, n (%)1 (4.0)	
Revascularization, n (%)	1 (4.0)
HF, n (%)	4 (16.0)
**Medications**	
ACEi/ARB, n (%)	5 (20.0)
Beta blockers, n (%)	4 (16.0)
Calcium channel blockers, n (%)	7 (28.0)
Phosphate binders,n (%)	1 (4.0)

HF: Heart Failure; ACEi/ARB: Angiotensin-Converting Enzyme inhibitors and Angiotensin II Receptor Blocker; BMI: Body Mass Index; BSA: Body Surface Area; DBP: diastolic blood pressure; HR: heart rate; MAP: mean arterial pressure; SBP: systolic blood pressure. Continuous data are expressed as mean (SD) or median (IQR) as appropriate, categorical as counts and percentages.

Echocardiographic parameters of the patients are provided in Table 2. The mean LV ejection fraction was 56.4 (10.9) %, and the mean LV end-diastolic and end-systolic volumes were 114.3 (32.2) and 51.1 (23.5) mL, respectively.

**Table 2:** Echocardiographic parameters of the patients.

LVESV, Mean (SD) mL	51.1 (23.5)
LVESVi, Mean (SD) mL/m^2^	28.9 (14.7)
LVEDV, Mean (SD) mL	114.3 (32.2)
LVEDVi, Mean (SD) mL/m^2^	64.4 (18.4)
LVEF, Mean (SD) %	56.4 (10.9)

LVESV, left ventricular end-systolic volume; LVESVi, left ventricular end-systolic volume indexed; LVEDV, left ventricular end-diastolic volume; LVEDVi, left ventricular end-diastolic volume indexed; LVEF, left ventricular ejection fraction.

### Inter-observer variability.

**Fig 3:**
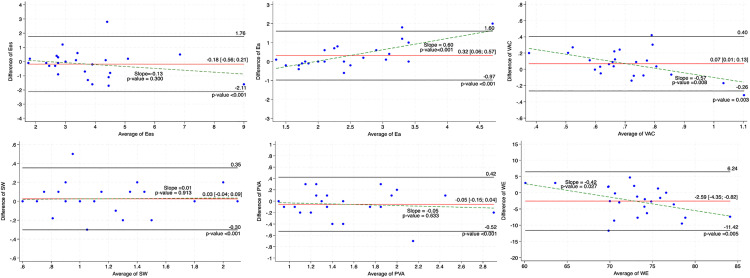
Bland-Altman plots for the of inter-observer variability in end-systolic elastance (Ees), effective arterial elastance (Ea), ventriculo-arterial coupling (VAC), stroke work (SW), pressure volume area (PVA), and work efficiency (WE).

Table 3 summarizes the inter- and intra-observer variabilities for the PV loop parameters. PVA and SW demonstrated excellent inter-observer agreement with ICC values of 0.95 (95% CI: 0.87; 0.98) and 0.96 (95% CI: 0.91; 0.98), respectively. Ea and Ees showed good agreement with ICC values of 0.79 (95% CI: 0.49; 0.91) and 0.90 (95% CI:0.77; 0.96), while VAC and WE exhibited moderate agreement 0.68 (95% CI: 0.23; 0.85) and 0.72 (95% CI:0.28; 0.89). All ICC values were statistically significant (p < 0.05) indicating acceptable agreement between observers. Bland-Altman plot analysis is represented in Fig 3 and in S1 Table. SW (bias = 0.03 ± 0.16; LoA: −0.30; 0.35; slope p = 0.913), PVA (bias = −0.05 ± 0.24; LoA:-0.52; 0.42; slope p = 0.633), and Ees (bias = −0.18 ± 0.96; LoA:-2.11; 1.76; slope p = 0.300) demonstrated minimal bias and non-significant proportional error, indicating high reproducibility. In contrast, Ea (bias = 0.32 ± 0.64; LoA:-0.97; 1.60), VAC (bias = 0.07 ± 0.16; LoA:-0.26; 0.40), and WE (bias = −2.59 ± 4.42; LoA:-11.42; 6.24) have statistically significant proportional bias (slope values = 0.60, −0.57, and −0.42, respectively; p-values <0.001, 0.008, and 0.027), indicating that the disagreement between observers increased with higher measurement values. The 95% CI for bias for all parameters included zero, indicating no systematic bias, except for WE, which showed a 95% CI of −4.35 to −0.82, suggesting a small but statistically significant bias between observers. Overall, inter-observer variability was low, with 96% of measurements (24 out of 25) falling within the limits of agreement for each parameter.

**Table 3:** Inter- and intra-observer variability for PV loop derived parameters.

Parameter	Inter-observer variability		Intra-observer variability	
	ICC (95% C.I.)	p-value	ICC (95% C.I.)	p-value
Ees	0.90 (0.77; 0.96)	<0.001	0.96 (0.90; 0.98)	<0.001
Ea	0.79 (0.49; 0.91)	<0.001	0.98 (0.94; 0.99)	<0.001
VAC	0.68 (0.23; 0.85)	=0.003	0.78 (0.51; 0.91)	<0.001
SW	0.96 (0.91; 0.98)	<0.001	0.98 (0.96; 0.99)	<0.001
PVA	0.95 (0.87; 0.98)	<0.001	0.98 (0.96; 0.99)	<0.001
WE	0.72 (0.28; 0.89)	=0.005	0.85 (0.65; 0.94)	<0.001

Ees: end-systolic elastance; Ea: arterial elastance; VAC: ventriculo-arterial coupling; PVA: pressure volume area; SW: stroke work; WE: work efficiency.

### Intra-observer variability.

**Fig 4:**
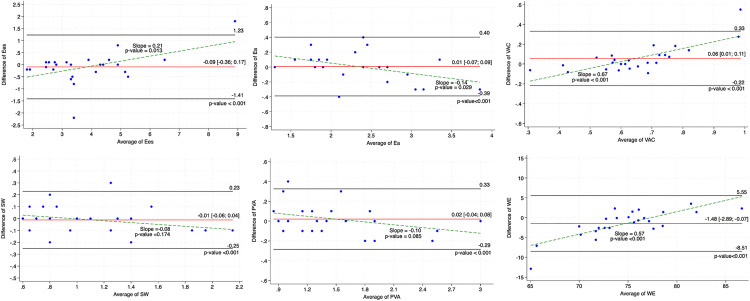
Bland-Altman plots for the of intra-observer variability in end-systolic elastance (Ees), effective arterial elastance (Ea), ventriculo-arterial coupling (VAC), stroke work (SW), pressure volume area (PVA), and work efficiency (WE).

The ICC analysis for the intra-observer variability recognized Ees, Ea, SW and PVA as having excellent agreement, with ICCs of 0.96 (95% CI: 0.90–0.98), 0.98 (95% CI: 0.94–0.99), 0.98 (95% CI: 0.96–0.99), and 0.98 (95% CI: 0.96–0.99), respectively (all p < 0.001), as summarized in Table 3. VAC and WE showed good levels of agreement, with ICCs of 0.78 (95% CI: 0.51–0.91; p < 0.001) and 0.85 (95% CI: 0.65–0.94; p < 0.001), respectively. Bland-Altman plots illustrating the agreement and bias for intra-observer measurements are shown in Fig 4, with corresponding statistical estimates provided in S1 Table. SW and PVA demonstrated mean differences of −0.01 ± 0.12 (LoA:-0.25; 0.23) and 0.02 ± 0.15 (LoA:-0.29; 0.33), respectively, and showed non-significant proportional bias with regression slopes of −0.10 (p = 0.085) and −0.08 (p = 0.174), indicating high reproducibility. In contrast, Ees (bias = −0.09 ± 0.66; LoA:-1.41; 1.23; slope = −0.14, p = 0.029), Ea (bias = −0.09 ± 0.19; LoA:-0.39; 0.40; slope = 0.21, p = 0.013), VAC (bias = 0.06 ± 0.14; LoA:-0.22; 0.33; slope = 0.57, p < 0.001), and WE (bias = −1.48 ± 3.52; LoA:-8.51; 5.55; slope = 0.67, p < 0.001) exhibited statistically significant proportional bias, indicating increasing disagreement with higher values. The 95% CI of the bias confirmed that all parameters demonstrated stable repeatability, while only WE showed a small systematic bias, with a 95% CI of −2.89 to −0.07. All parameters demonstrated acceptable agreement, with 100% of measurements falling within the limits of agreement, supporting the robustness of intra-observer reproducibility across all PV loop-derived parameters.

### Sample size estimates.

**Fig 5:**
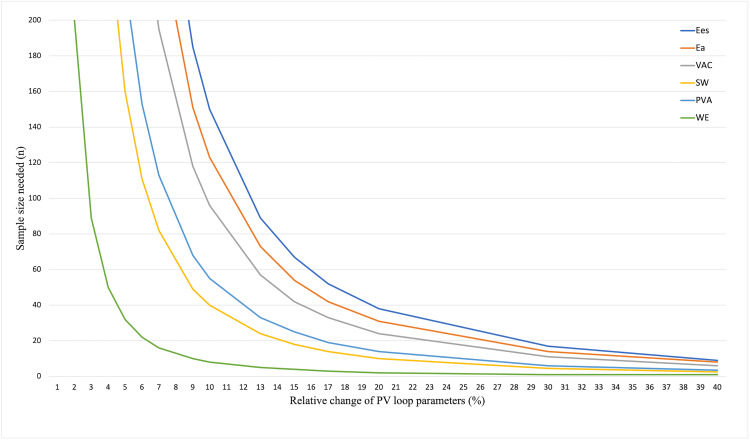
Sample size calculation for the PV loop parameters. Number of the patients needed for end-systolic elastance (Ees), effective arterial elastance (Ea), ventriculo-arterial coupling (VAC), stroke work (SW), pressure volume area (PVA), and work efficiency (WE) to detect the relative percent change in mean values.

Fig 5 demonstrates the sample sizes needed to detect 10%, 15%, and 20% relative changes of the PV loop derived parameters. According to Table 4, 150, 123, 96, 40, 55, and 8 patients are required to detect 10% changes for Ees, Ea, VAC, SW, PVA, and WE, respectively. WE showed the lowest sample size to detect changes for 10%, demonstrating the highest sensitivity. A sample of 24 patients is sufficient to accurately detect a 20% relative change in VAC.

**Table 4:** Sample size calculation for the PV loop parameters to detect 10, 15, and 20% relative change compared to the mean values of the first reader with 90% power and α error of 0.05.

Parameter	Mean difference ±	SD Sample size (n)		
		10%	15%	20%
Ees	−0.18 ± 0.97	150	67	38
Ea	0.32 ± 0.64	123	54	31
VAC	0.07 ± 0.16	96	42	24
SW	0.03 ± 0.16	40	18	10
PVA	−0.05 ± 0.24	55	25	14
WE	−2.59 ± 4.42	8	4	2

Ees, end-systolic elastance; Ea, effective arterial elastance; VAC, ventriculo-arterial coupling; PVA, pressure volume area; SW, stroke work; WE, work efficiency.

## Discussion

In a HD cohort we found excellent intra-observer reliability for Ees, Ea, SW and PVA, with good intra-observer reliability for VAC and WE, while inter-observer agreement was excellent for SW and PVA, good for Ees and Ea, and moderate for VAC and WE. Bland–Altman analysis for inter-observer agreement confirmed these findings: Ees, SW and PVA showed minimal bias and no significant proportional error, whereas Ea, VAC and WE displayed significant proportional bias, indicating value-dependent disagreement between readers. The data on the Bland-Altman plots were within LoA, suggesting the lack of disagreement between the observers. This displays the statistical significance of the reliability and thus, the applicability of the analysis results in clinically and research relevant settings.

Our results are consistent with previous reports. A study of 285 patients undergoing HD by Zuo et al. [20] have shown that VAC is a reliable tool to assess cardiac function. It confirms the data of our study, and the p-values (p = 0.003 for inter- and p < 0.001 for intra-observer variability) strengthens the statistical significance of the results. Similarly, in a longitudinal cohort study of breast cancer patients, Narayan et al. reported acceptable intra- and inter- observer reproducibility for Ea/Ees, aligning with our findings of good to excellent reproducibility for elastance based parameters [21]. Reproducibility of an alternative (centroid distance) PV loop parameter was assessed by Cioffi et al. in patients with stable HF [22]. The study reports at least a good level of reproducibility from ICC analysis of 30 patients, which is consistent with our findings of moderate to excellent level of ICC. Similarly, Monosillo et al. showed that the variability of PV loop derived parameters for the non-invasive diagnostics of the cardiac patients was acceptable based on ICC analysis [23]. These results support our observation that most PV loop indices, in particular PVA and SW, are highly reproducible, while VAC and WE show comparatively lower inter-observer agreement.

PV loop parameters help characterize global cardiovascular mechanics by integrating contractility, diastolic performance, and loading conditions. This approach offers a detailed view of cardiac function in both healthy individuals and patients on chronic therapy [9]. In clinical practice, TTE based assessment of Ees, Ea, VAC, SW, PVA, and WE enables serial monitoring of cardiac function, giving the capability of early identification of a subclinical dysfunction and assessment of the response to medical interventions. This facilitates the optimized treatment strategies and contributes to a comprehensive prognostic evaluation. Our findings indicate that parameters such as Ees, PVA, and SW are robust and reliable for both research and clinical use, even across observers, while Ea, VAC, and WE may require additional standardization or cautious interpretation, particularly at lower or higher value ranges. Standardization of measurement protocols and training may help to reduce variability in these indices. Previous studies by Seemann et al. [11] and Edlund et al. [10] have evaluated the reproducibility of noninvasive PVloop–derived indices in patients with various forms of cardiac dysfunction, particularly HF. Seemann et al. demonstrated the feasibility of PVloop estimation from echocardiography in cardiac patients, while Edlund et al. extended this work by assessing reproducibility across different subgroups of HF with preserved, reduced, and mildly reduced ejection fraction.

The present study is, to our knowledge, the first to investigate the reliability of these parameters in patients undergoing maintenance HD. This population presents unique hemodynamic challenges due to frequent and pronounced shifts in preload and afterload during dialysis sessions. Evaluating PV-loop parameters under these conditions allowed us to test the robustness of the method in a setting characterized by substantial cardiovascular variability. Therefore, our findings provide novel evidence supporting the applicability and reproducibility of echocardiography-derived PVloop parameters in a clinically relevant, yet previously understudied, patient group.

Sample size analysis indicated that VAC required the lowest number of patients to detect clinically relevant changes, with 24 patients being sufficient to capture a 20% change. This suggests that VAC, despite its moderate inter-observer agreement, may still represent an efficient endpoint in clinical studies.

A major strength of our study is the methodological approach, including blinded independent assessments and dual assessments approaches (the use of ICC and Bland-Altman analysis), which together provides a rigorous evaluation of both inter- and intra-observer reliability. However, the study also has some limitations. It is a single-center, subgroup analysis from a larger ongoing trial, which may limit the generalisability of the findings. Patients were selected randomly, which resulted in uneven gender distribution, however, a sensitivity analysis stratified by sex showed comparable results, suggesting that the gender distribution did not influence the main findings (S2 Table). Moreover, the reproducibility may vary based on the level of training of the interpreters. In our laboratory, joint reading sessions were implemented to standardize the methodology of image analysis. Our findings suggest that agreement between observers depends on the magnitude of the measurement: values at the lower or higher end of the spectrum are more prone to disagreement. This indicates that while SW, PVA, and Ees can be interpreted with high confidence, caution is warranted when comparing VAC, Ea, and WE values across different readers or when following patients longitudinally.

An important clinical implication of our findings is that longitudinal or interventional studies using PVloop–derived parameters must ensure that observed changes exceed the intrinsic measurement variability of each parameter. Only changes greater than this variability can be interpreted as clinically meaningful. Furthermore, the variability estimates provided here can serve as quantitative inputs for sample-size calculation in future clinical studies, improving methodological rigor in trials using PVloop–derived endpoints. Taken together, these results advance current knowledge by demonstrating that PVloop parameters can be reliably measured in HD patients using a fully noninvasive approach. Their application may support individualized cardiovascular assessment, improve monitoring strategies, and enhance the design of future studies exploring cardiac function in dialysis-dependent populations.

## Conclusions

This study demonstrates the reliability of PV loop parameters derived from TTE imaging, with particularly strong reproducibility for Ees, PVA and SW. Ea, VAC and WE showed moderate inter-observer agreement and value-dependent variability, underscoring the need for cautious interpretation. The analysis was also strengthened by the limited bias and acceptable agreement using the Bland-Altman plot. Overall, our findings support the use of selected PV loop parameters in both clinical and research purposes in patients receiving HD.

## Supporting information

S1 Table Bland-Altman plots. Interobserver variability of PV loop parameters performed by two observers, and intraobserver variability performed by one observer within 1 week interval.
(DOCX)

S2 Table Sensitivity analysis of inter- and intra-observer variability stratified by gender. (DOCX)

S1 File Data underlying the findings described in the manuscript. (XLSX)
