# Supplementary material for: Reliability of pressure-volume loop parameters derived from transthoracic echocardiography in patients undergoing hemodialysis
Source: PLoS One. 2026 Jan 5;21(1):e0340206. doi: 10.1371/journal.pone.0340206 (PMC12768255; doi:10.1371/journal.pone.0340206)
Supplement: S1 Table — Interobserver variability of PV loop parameters performed by two observers, and intraobserver variability performed by one observer within 1 week interval. (DOCX) [file pone.0340206.s001.docx]

**S1 Table.** Bland-Altman plots: Interobserver variability of PV loop parameters performed by two observers, and intraobserver variability performed by one observer within 1 week interval.

Inter-OV(n=25) Intra-OV (n=25)

PV loop parameters

End-systolic elastance (Ees) -0.18 ± 0.96 -0.09 ± 0.66

95% CI [-0.56; 0.21] 95% CI [-0.36; 0.17]

LoA [ -2.11; 1.76] LoA[-1.41; 1.23]

Arterial elastance (Ea) 0.32 ± 0.64 0.01 ± 0.19

95% CI [0.06; 0.58] 95% CI [-0.07; 0.09]

LoA [-0.97; 1.60] LoA [-0.39; 0.40]

Ventriculo-arterial 0.07 ± 0.16 0.06 ± 0.14

coupling (VAC) 95% CI [0.00; 0.14] 95% CI [0.00; 0.12]

LoA [-0.26; 0.40] LoA [-0.22; 0.33]

Stroke work (SW) 0.03 ± 0.16 -0.01 ± 0.12

95% CI [-0.04; 0.09] 95% CI [-0.06; 0.04]

LoA [-0.30; 0.35] LoA [-0.25; 0.23]

Pressure volume area (PVA) -0.05 ± 0.24 0.02 ± 0.15

95% CI [-0.15; 0.04] 95% CI [-0.04; 0.08]

LoA [-0.52; 0.42] LoA [-0.29; 0.33]

Work efficiency (WE) -2.59 ± 4.42 -1.48 ± 3.52

95% CI [-4.35; -0.82] 95% CI [-2.89; -0.07]

LoA [-11.42; 6.24] LoA [-8.51; 5.55]

Values presented as bias (mean difference) ± SD and LoA [95% CI].
