# Supplementary material for: Reliability of pressure-volume loop parameters derived from transthoracic echocardiography in patients undergoing hemodialysis
Source: PLoS One. 2026 Jan 5;21(1):e0340206. doi: 10.1371/journal.pone.0340206 (PMC12768255; doi:10.1371/journal.pone.0340206)
Supplement: S2 Table — (DOCX) [file pone.0340206.s002.docx]

**S2 Table.** Sensitivity analysis of inter- and intra-observer variability stratified by gender.

*Inter-observer variability*

Parameter Male ICC(95% CI) p-value Female ICC (95% CI) p-value

Ees 0.80 (0.48–0.92) <0.001 0.91 (0.42–0.99) 0.011

Ea 0.82 (0.57–0.93) <0.001 0.72 (0.38–0.88) 0.012

VAC 0.66 (0.24–0.84) 0.008 0.61 (0.20–0.81) 0.015

PVA 0.96 (0.89–0.98) <0.001 0.93 (0.82–0.97) <0.001

SW 0.97 (0.93–0.99) <0.001 0.94 (0.88–0.98) <0.001

WE 0.71 (0.26–0.89) 0.003 0.86 (0.11–0.98) 0.013

*Intra-observer variability*

Parameter Male ICC(95% CI) p-value Female ICC (95% CI) p-value

Ees 0.93 (0.81–0.97) <0.001 0.95 (0.65–0.99) 0.002

Ea 0.97 (0.92–0.98) <0.001 0.97 (0.82–0.99) <0.001

VAC 0.77 (0.42–0.91) 0.001 0.85 (0.28–0.98) 0.026

PVA 0.98 (0.91–0.98) <0.001 0.82 (0.26–0.97) 0.040

SW 0.96 (0.91–0.98) <0.001 0.89 (0.25–0.98) 0.015

WE 0.83 (0.57–0.94) <0.001 0.92 (0.39–0.98) 0.009
